# Supplementary material for: High-Sensitivity Dual Electrochemical QCM for Reliable Three-Electrode Measurements
Source: Sensors (Basel). 2021 Apr 7;21(8):2592. doi: 10.3390/s21082592 (PMC8068083; doi:10.3390/s21082592)
Supplement: Supplementary file 1 [file sensors-21-02592-s001.pdf]

Supplementary information for

High-sensitivity dual electrochemical QCM for reliable three-electrode measurements

Dávid Tóth[1,2], Manuel Kasper[1], Ivan Alic[2], Mohamed Awadein[1,2], Andreas Ebner[2], Doug Baney[3], Georg Gramse[1,4] and Ferry Kienberger[1]

1. Keysight Technologies GmbH, Linz 4020, Austria; david.toth@jku.at (D.T.); manuel.kasper@keysight.com (M.K.); mohamed.awadein@keysight.com (M.A.); georg.gramse@jku.at (G.G.)

2. Applied Experimental Biophysics, Johannes Kepler University, Linz 4020, Austria; ivan.alic@jku.at (I.A.); andreas.ebner@jku.at (A.E.)

3. Keysight Technologies Inc., Santa Clara 95052, USA, doug_baney@keysight.com

4. Molecular Biophysics and Membrane Physics, Johannes Kepler University, Linz 4020, Austria;

Correspondence: ferry_kienberger@keysight.com

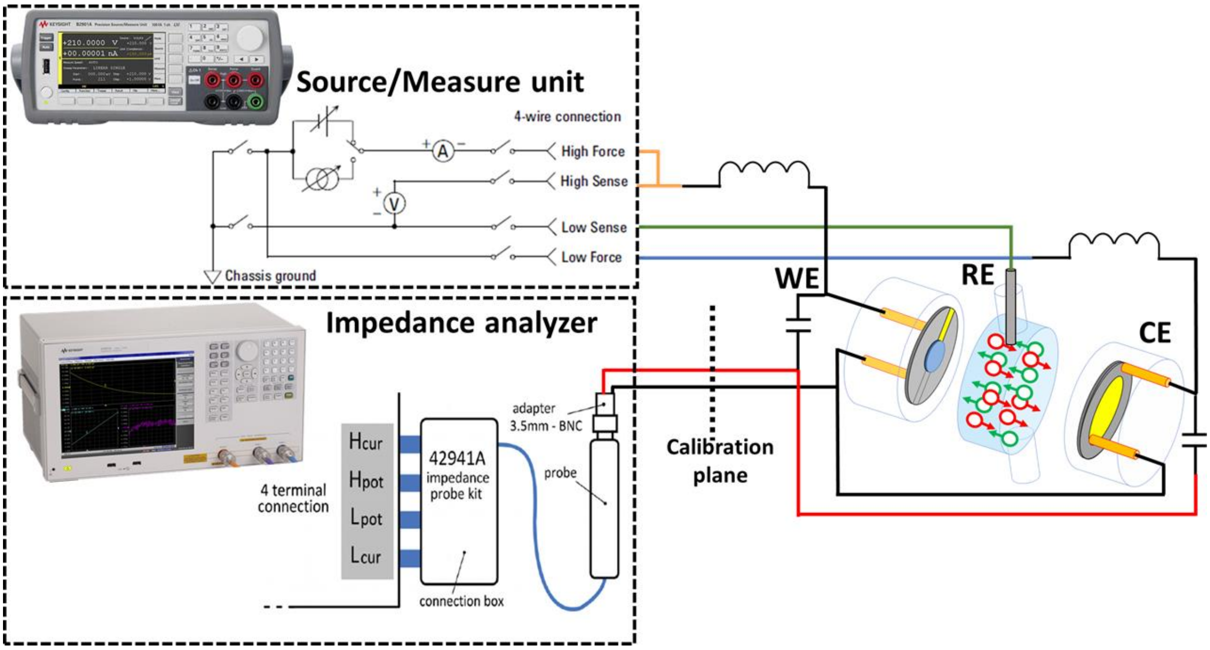

Figure S1. *Detailed connection scheme of the presented dual-EC-QCM setup.*

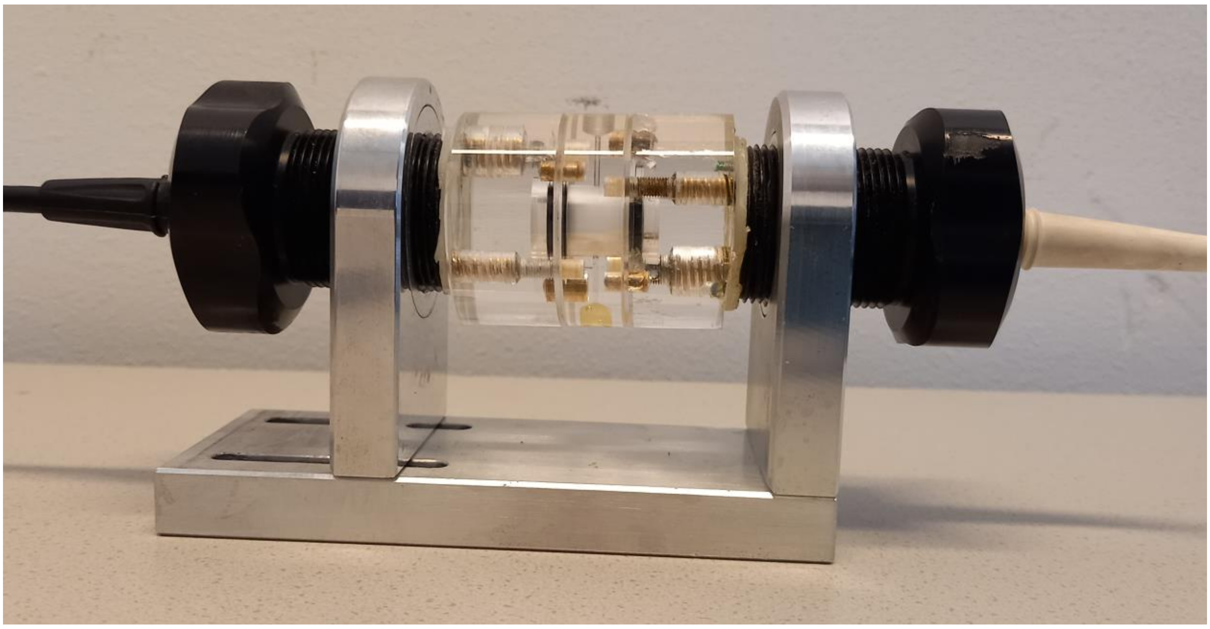

Figure S2. *Photo of the modified QCM cell that accommodates a quartz sensor on each side. Electrical connection to the crystals is done via spring contacts that are in direct connection with BNC ports. Volume of the liquid compartment is ~1 ml.*

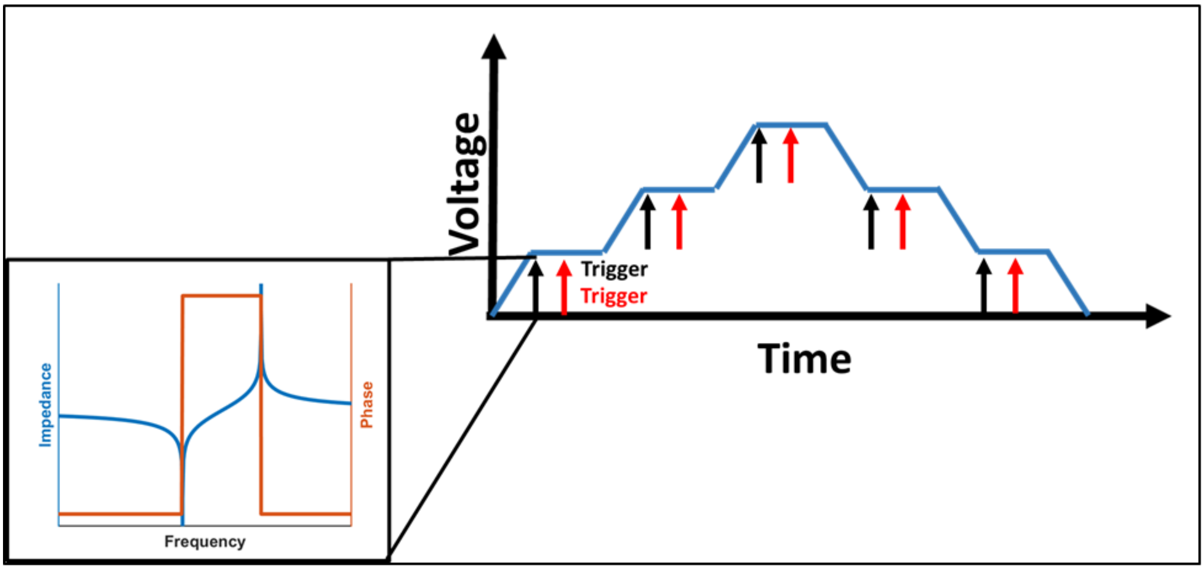

Figure S3. *Workflow of the combined cyclic voltammetry and impedance spectroscopy measurements. The voltage between the two crystals is ramped up stepwise as shown. The current is measured while the voltage is ramped. At each step the voltage is kept constant for a short time while the frequency sweep for both QCMs are done by the impedance analyzer. The trigger signal of the WE and CE crystals is indicated by the black and red arrows respectively.*

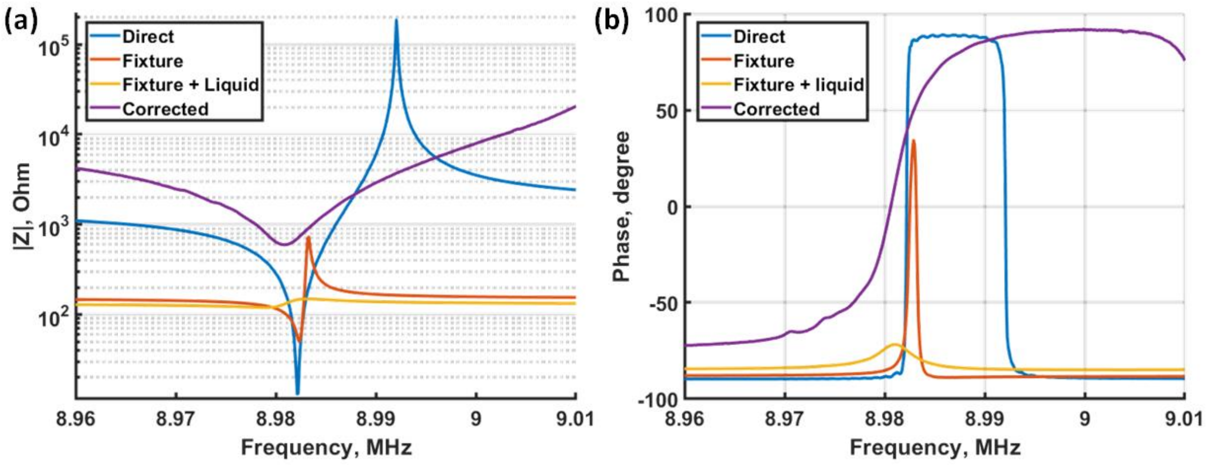

Figure S4. *Comparison of the complex impedance of the QCM crystal under four different conditions. Magnitude (a) and phase (b) of the complex impedance of the QCM crystal. First, we show the response with direct connection to the impedance probe in air, then the response of the QCM connected via the fixture, the response of the QCM in distilled water connected via the fixture and lastly under the latter conditions and the applied correction. The responses in the different conditions are represented by the blue, orange and yellow lines respectively.*

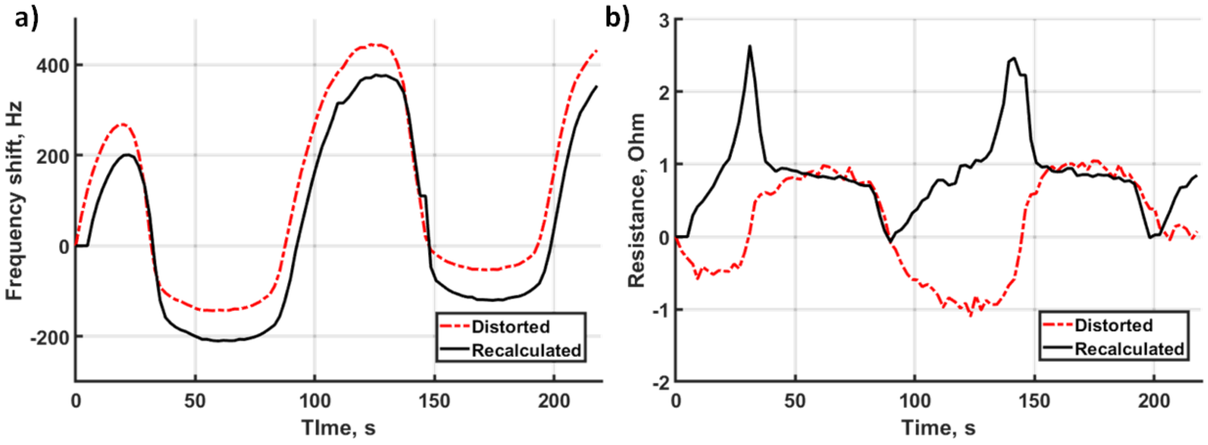

Figure S5. *Effect of complex impedance correction. (a) Frequency shift during CV before (red) and after correction (black) (b) Resistance change during CV before (red) and after (black) correction*

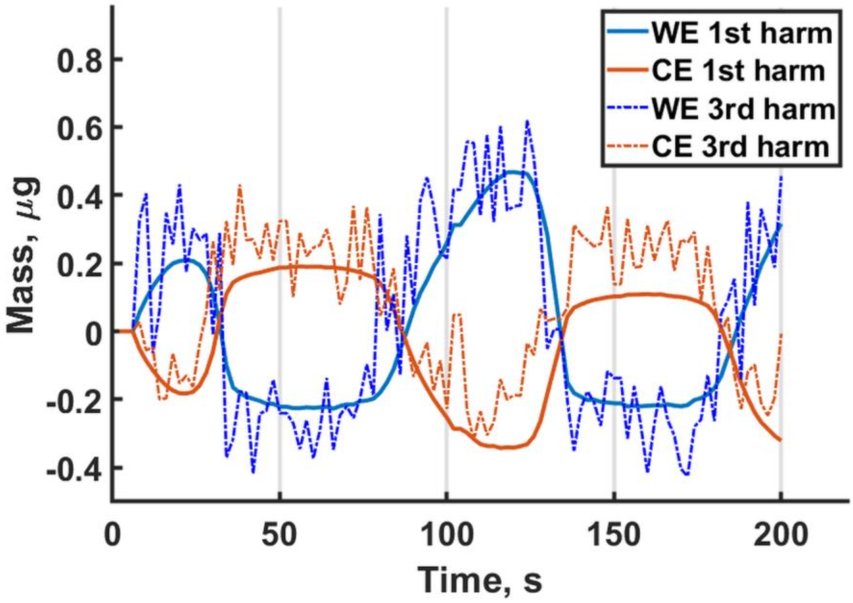

Figure S6. *Mass deposition calculated from consequent first and third harmonic detection measurements for both WE and CE.*

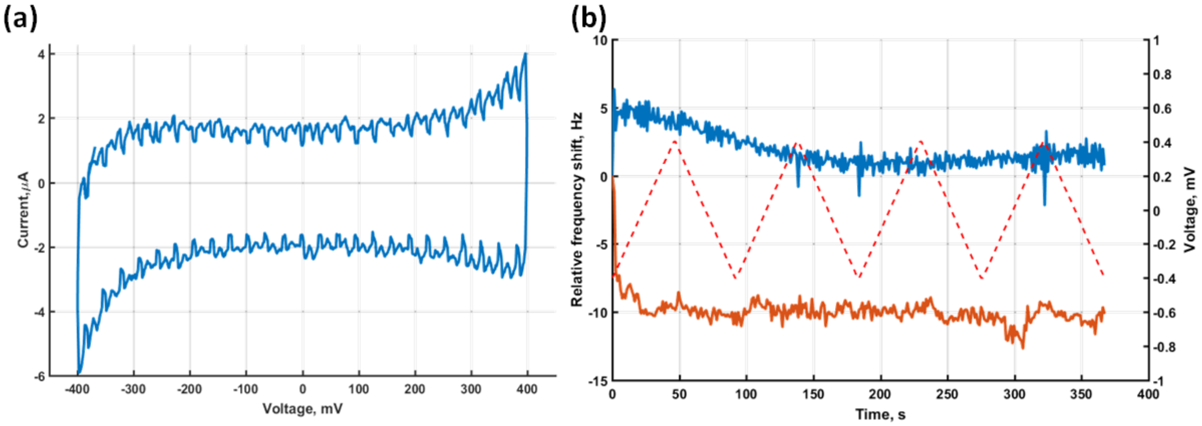

Figure S7. *CV and QCM response in aqueous 0.2 M H_2SO_4. (a) Featureless cyclic voltammogram recorded on the -0.4 V to 0.4 V voltage range. (b) Resonance frequency change of the WE (blue curve) and CE (orange curve) and change of potential difference (dashed line) during several CV cycles. Scanning speed was 20 mV/s.*

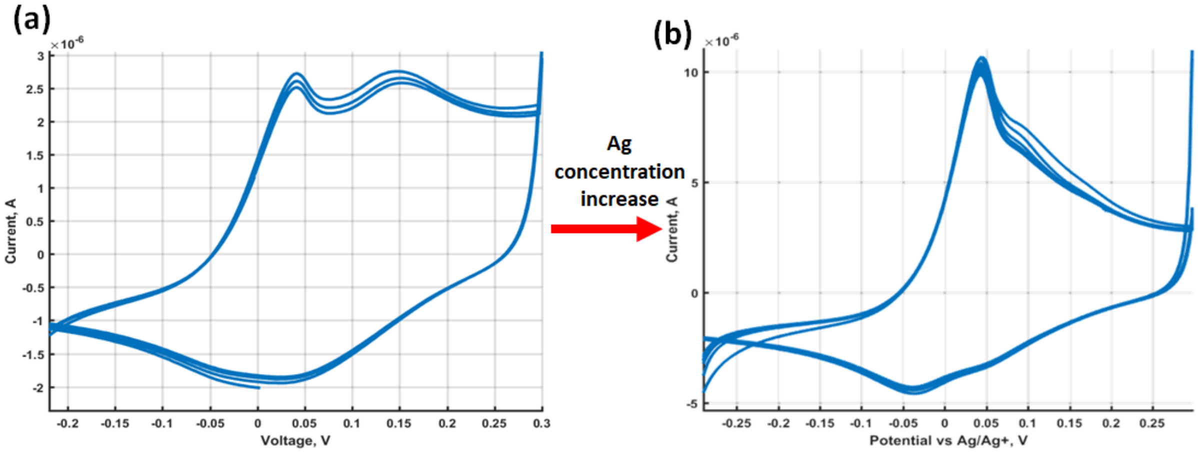

Figure S8. *Identification of underpotential deposition peaks of Ag on Au. Oxidation peak found at ~0.04 V vs Ag, reduction peak found at -0.04 V vs Ag. (a) Cyclic voltammogram in aqueous 0.2 M H_2SO_4, 10^{-3} M Ag_2SO_4. (b) Cyclic voltammogram in aqueous 0.2 M H_2SO_4, $2x10^{-3}$ M Ag_2SO_4. We note that the additional CV features (e.g. oxidation peak at 0.15 V) in panel (a) are also Ag redox peaks of interest. Due to the polycrystalline nature of the Au films on the employed sensors variation of the relative peak heights and position was observed across various QCM sensors.*

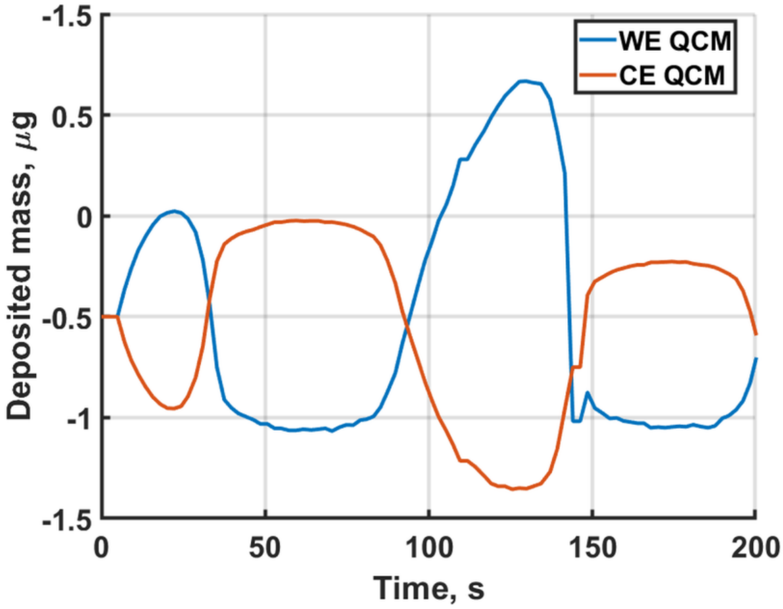

Figure S9. *Mass deposition calculated using Sauerbrey's relation for the working electrode QCM and counter electrode QCM represented by the blue and orange curves respectively.*
